# Haemoglobin degradation underpins the sensitivity of early ring stage *Plasmodium falciparum* to artemisinins

**DOI:** 10.1242/jcs.178830

**Published:** 2016-01-15

**Authors:** Stanley C. Xie, Con Dogovski, Eric Hanssen, Francis Chiu, Tuo Yang, Maria P. Crespo, Che Stafford, Steven Batinovic, Silvia Teguh, Susan Charman, Nectarios Klonis, Leann Tilley

**Affiliations:** 1 Department of Biochemistry and Molecular Biology, The University of Melbourne, Melbourne, Victoria 3010, Australia; 2 Advanced Microscopy Facility, Bio21 Molecular Science and Biotechnology Institute, The University of Melbourne, Melbourne, Victoria 3010, Australia; 3 Monash Institute of Pharmaceutical Sciences, Faculty of Pharmacy and Pharmaceutical Sciences, Melbourne, Victoria 3010, Australia; 4 Department of Microbiology, University of Valle, 13 #100-00, Cali, Valle del Cauca, Colombia; 5 Department of Biomedical Sciences, Santiago de Cali University, 25, Cali, Valle del Cauca, Colombia; 6 Walter+Eliza Hall Institute, Department of Medical Biology, The University of Melbourne, Parkville, Victoria 3052, Australia

**Keywords:** Malaria, Plasmodium, Artemisinin, Haemoglobin degradation, Falcipains, Resistance

## Abstract

Current first-line artemisinin antimalarials are threatened by the emergence of resistant *Plasmodium falciparum*. Decreased sensitivity is evident in the initial (early ring) stage of intraerythrocytic development, meaning that it is crucial to understand the action of artemisinins at this stage. Here, we examined the roles of iron (Fe) ions and haem in artemisinin activation in early rings using Fe ion chelators and a specific haemoglobinase inhibitor (E64d). Quantitative modelling of the antagonism accounted for its complex dependence on the chemical features of the artemisinins and on the drug exposure time, and showed that almost all artemisinin activity in early rings (>80%) is due to haem-mediated activation. The surprising implication that haemoglobin uptake and digestion is active in early rings is supported by identification of active haemoglobinases (falcipains) at this stage. Genetic down-modulation of the expression of the two main cysteine protease haemoglobinases, falcipains 2 and 3, renders early ring stage parasites resistant to artemisinins. This confirms the important role of haemoglobin-degrading falcipains in artemisinin activation, and shows that changes in the rate of artemisinin activation could mediate high-level artemisinin resistance.

## Abstract (precis)

**Summary:** Down-modulation of the expression of haemoglobin-degrading falcipains in *P. falciparum* renders early ring stage parasites resistant to artemisinins.

## INTRODUCTION

Malaria is a debilitating parasitic disease caused by protozoan parasites of the genus *Plasmodium*. Annually, about 200 million new infections of *P. falciparum* malaria are established, causing about 584,000 deaths (World Health Organization, World Malaria Report, 2014). Mortality and morbidity are associated with the asexual blood stage of the lifecycle of the parasite, and most chemotherapeutic agents target this phase. Most countries with endemic malaria have adopted the World Health Organization (WHO)-recommended artemisinin-based combination therapies (ACTs) for treating malaria (Enserink, 2010).

Artemisinin (or Qinghaosu, QHS) is a sesquiterpene lactone with a 1,2,4-trioxane core incorporating an endoperoxide linkage that is essential for activity (O'Neill et al., 2010). QHS and its derivatives [collectively referred to as artemisinin(s) or ART(s)] clear *P. falciparum* infections rapidly, providing prompt therapy for both uncomplicated and severe infections (White et al., 2014). A disadvantage of the clinically relevant lactol derivative, dihydroartemisinin (DHA), is its short (∼2 h) *in vivo* half-life, which necessitates a multi-dose treatment regimen (White et al., 2014). The mechanism of action of ARTs remains poorly understood, but it is widely assumed that they need to be activated by reductive cleavage of the endoperoxide ring mediated by iron (Fe) ions (Li and Zhou, 2010). The activated intermediates are thought to react with nucleophilic groups within parasite proteins and other cellular components, leading to parasite killing (Li and Zhou, 2010).

There is debate as to the nature of the Fe-based activator. Cells maintain a low steady-state labile Fe pool. Additional Fe-containing species are generated when trophozoites take up and digest host haemoglobin (Abu Bakar et al., 2010). Although most of the haem is sequestered as haemozoin (Egan, 2008), a fraction might escape detoxification to form a haem-Fe pool (Ginsburg et al., 1998; Loria et al., 1999). Additional labile Fe might be released by degradation of haem in the digestive vacuole (Loria et al., 1999) or the parasite cytoplasm (Ginsburg et al., 1998).

A distinguishing feature of ARTs is their parasiticidal activity at all stages of intraerythroctyic development (Klonis et al., 2013b). This includes the young (ring stage) form of the parasite, which is resistant to most other chemotherapeutic agents. Using a pulsed drug exposure assay that mimics the profile of exposure *in vivo*, we have previously shown that the action of ARTs against mature stage parasites (trophozoites) requires the activity of the parasite falcipain (FP) family of haemoglobinases (Klonis et al., 2011). ARTs are less active against the mid-ring stage of infection, consistent with a lower level of haemoglobin digestion at this stage. However, this simple view of activation of ARTs does not explain the very high activity of ARTs against the early ring stage (2–4 h post-invasion) of laboratory strains of *P. falciparum* (Klonis et al., 2013b).

Understanding the mechanism of action of ARTs in early rings is important given the recent emergence of resistance to ARTs in South-East Asia (Tun et al., 2015). This manifests as slowed parasite clearance in patients, which correlates with decreased *in vitro* drug sensitivity of the youngest rings (Witkowski et al., 2013). In this work, we present a detailed analysis of the roles of free Fe and haem in the activity of ARTs at different stages of intraerythrocytic development. We show that a falcipain-generated activator is crucial for activation of ARTs in early rings and that delayed expression of falcipain activity renders early rings resistant to ARTs.

## RESULTS

### Characterisation of Fe chelators

Fe chelators have been shown to antagonise the activity of ARTs in standard assays (where drug pressure is maintained throughout the parasite lifecycle) (Eckstein-Ludwig et al., 2003), and in trophozoites in stage-specific drug assays (Stocks et al., 2007). Desferrioxamine (DFO) has been employed in such studies, but its low membrane permeability makes it unsuitable for pulse assays involving ring stage parasites (Lytton et al., 1993). We therefore examined the suitability of alternative Fe chelators.

**Fig. 1.**
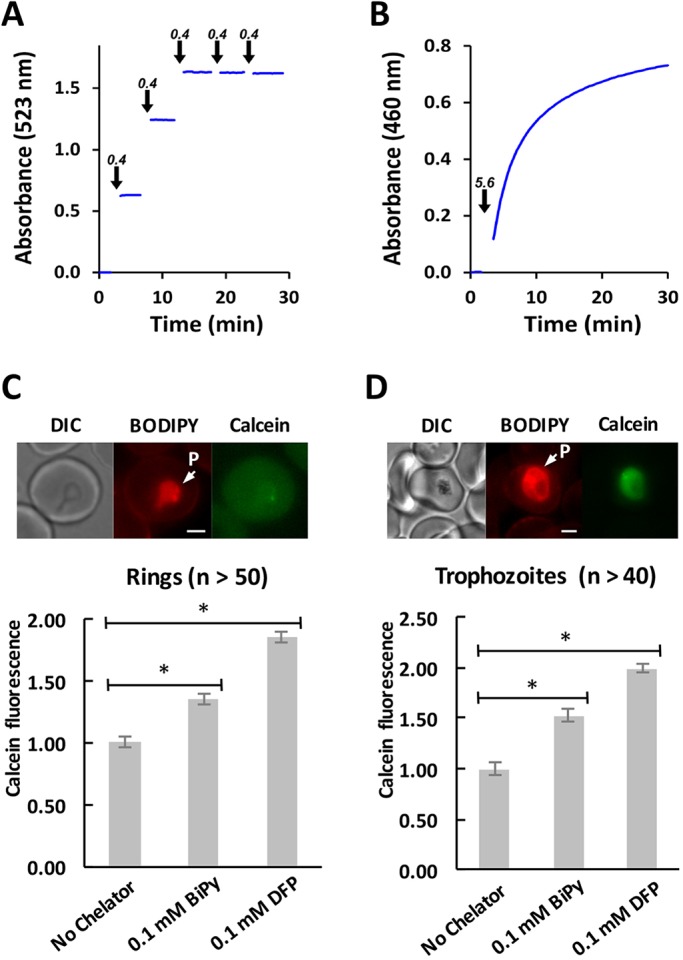
**Characterisation of Fe chelators.** (A,B) The rate of Fe chelation by DFP is slower than chelation by BiPy at pH 5. The absorbance of a solution of FeSO_4_ (150 µM in 20 mM MES, pH 5) was monitored over time. BiPy (A) or DFP (B) were added at the times indicated by the arrows. The concentration of chelator added at each time point is also indicated above the arrow and is expressed as the stoichiometric equivalent for generating the Fe(chelator)_3_ complexes, taking into account the bidentate nature of the chelators. The measurements shown are representative of three experiments. (C,D) BiPy and DFP rapidly chelate intraparasitic Fe in ring and trophozoite stage parasites*.* Rings (5–15 h post-invasion) and trophozoites (28–38 h post-invasion) were labelled with BODIPY-TR-ceramide (red) and calcein-AM (green) and then incubated with BiPy for 15 min or DFP for 30 min. Shown are representative images of ring (C) and trophozoite (D) stage parasites showing the parasite (P) compartment. The intensity of the calcein signal within the parasite (P) compartment was quantified by live-cell imaging. Calcein fluorescence was normalized based on the untreated control. Error bars represent s.e.m. (*n*>40). *P<0.0001 for *t-*test with Welch's correction. Scale bars: 2 µm.

The time-dependent degradation of QHS *in vitro* in the presence of Fe can be monitored by liquid-chromatography mass spectrometry (LCMS) and represents a surrogate measure of Fe-mediated activation of QHS (Creek et al., 2005). These measurements were included to provide a qualitative indication of the impact of Fe chelators on QHS degradation. In the absence of chelators, QHS degrades with a half-life of ∼10 h in the presence of 3 mM FeSO_4_ at pH 5 (Table S1). We found that Fe-mediated opening of the QHS endoperoxide ring can be enhanced or inhibited by Fe chelators, and depends on the order of addition of reagents. Deferiprone (DFP), which shows high specificity for Fe^3+^ over Fe^2+^ under acidic conditions (Merkofer et al., 2006), substantially promoted Fe-mediated QHS degradation when FeSO_4_ was added to a solution containing DFP and QHS (Table S1, post-mix). However, when DFP and FeSO_4_ were pre-mixed prior to the addition of QHS, the rate of QHS degradation decreased with increasing pre-mixing times so that efficient quenching of QHS degradation was obtained after a 30 min pre-mix (half-life >50 h, Table S1; pre-mix). In contrast to DFP, the Fe^2+^-specific chelator 2,2′-bipyridyl (BiPy) required no pre-mixing with Fe to efficiently quench QHS degradation (half-life >50 h, Table S1). Spectrophotometric analysis of the chelator–Fe interactions demonstrated that BiPy chelates Fe rapidly (a time scale of seconds) and stoichiometrically, whereas DFP chelation occurs on a time scale of tens of minutes at pH 5 (Fig. 1A,B). Thus both chelators are expected to effectively inhibit Fe-mediated activation of QHS provided that the Fe is stoichiometrically chelated. For cellular measurements involving DFP, the DFP should be pre-incubated with cells to allow chelation to proceed to completion.

We next examined the ability of the chelators to deplete intracellular Fe levels in ring and trophozoite stage parasites. We used calcein-AM as a membrane-permeable probe whose fluorescence is quenched upon Fe^2+^ binding (Breuer et al., 1995; Epsztejn et al., 1997). The calcein fluorescence signal increased within 30 min of the addition of 100 μM BiPy or DFP (Fig. 1C,D) indicating that externally added BiPy and DFP are able to chelate free Fe inside parasites on a time scale of 30 min. This is consistent with previous reports showing that BiPy and DFP are able to deplete the labile Fe pool in *P. falciparum* (Clark et al., 2013).

### Fe chelators have limited effect on the activity of ARTs

BiPy and DFP were used to examine the role of Fe in activation of ARTs at different stages of intraerythrocytic development. Parasite cultures exposed to DFP and BiPy for 3 days exhibited 50% lethal dose (LD_50_) values of 92±20 µM and 48±16 µM, respectively (mean±s.d., *n*=4), consistent with previous reports (Pattanapanyasat et al., 1997; van Zyl et al., 1992). The toxicity of the chelators can be ascribed to their effects on the trophozoite stage, which is sensitive to short pulses of chelators [LD_50_ (6 h) values of ∼200 µM for both DFP and BiPy]. In contrast, early ring (1–3 h post-invasion) and mid-ring (7–10 h post-invasion) stage parasites were unaffected by exposure to the chelators for 6 h, at concentrations up to 1 mM (data not shown).

**Fig. 2.**
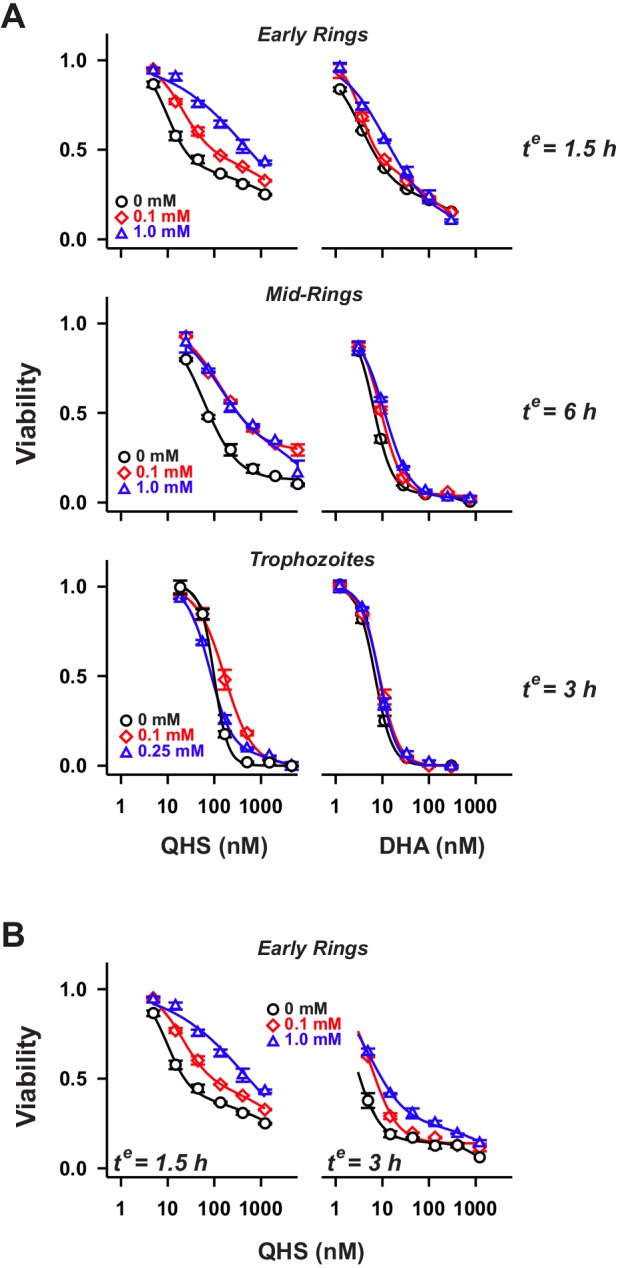
**Influence of labile Fe pool on the activity of ARTs at different parasite stages.** (A) Parasites at early ring, mid-ring and trophozoite stages (1–2 h, 7–8 and 24 h post-invasion, respectively) were pre-treated with BiPy at the concentrations indicated, and exposed to QHS or DHA for the exposure times (*t^e^*) indicated. Viability was assessed after 72 h. (B) Influence of exposure time on BiPy antagonism of QHS activity in early ring stage parasites. Error bars correspond to the range of technical duplicates in a typical experiment. Results from a number of experiments are summarised in Fig. 4D (symbols).

We previously demonstrated that LD_50_ values for ARTs are affected by the chemical features of the drug (i.e. QHS versus DHA), the parasite stage and the duration of the drug pulse (Klonis et al., 2013b). BiPy exerted modest antagonistic effects that were not only dependent on these parameters but also on the BiPy concentration (Fig. 2). For a given drug exposure time, BiPy exerted greater antagonism against QHS (Fig. 2A, left panels) than DHA (right panels) across all intraerythrocytic stages. The largest effect of BiPy was observed in early ring stage parasites subjected to a short (1.5 h) QHS pulse [23-fold increase in LD_50_ (1.5 h) with 1 mM BiPy; Fig. 2A]. This level of antagonism decreased with longer QHS exposure times [3-fold increase in LD_50_(3 h); Fig. 2B]. In contrast, BiPy only had a modest effect on DHA activity even at the shortest drug exposure times (2–3-fold increase in LD_50_ values; Fig. 2A and data not shown). Similar effects were observed with the Fe^3+^ chelator, DFP (Fig. S1A,B). All of these antagonistic effects are moderate, indicating that although free Fe might contribute to activation of ARTs, other activators and/or mechanisms of action are more important. We therefore explored the role of haemoglobin digestion products in activation of ARTs in ring stage parasites.

### Inhibition of cysteine proteases antagonises action of ARTs

**Fig. 3.**
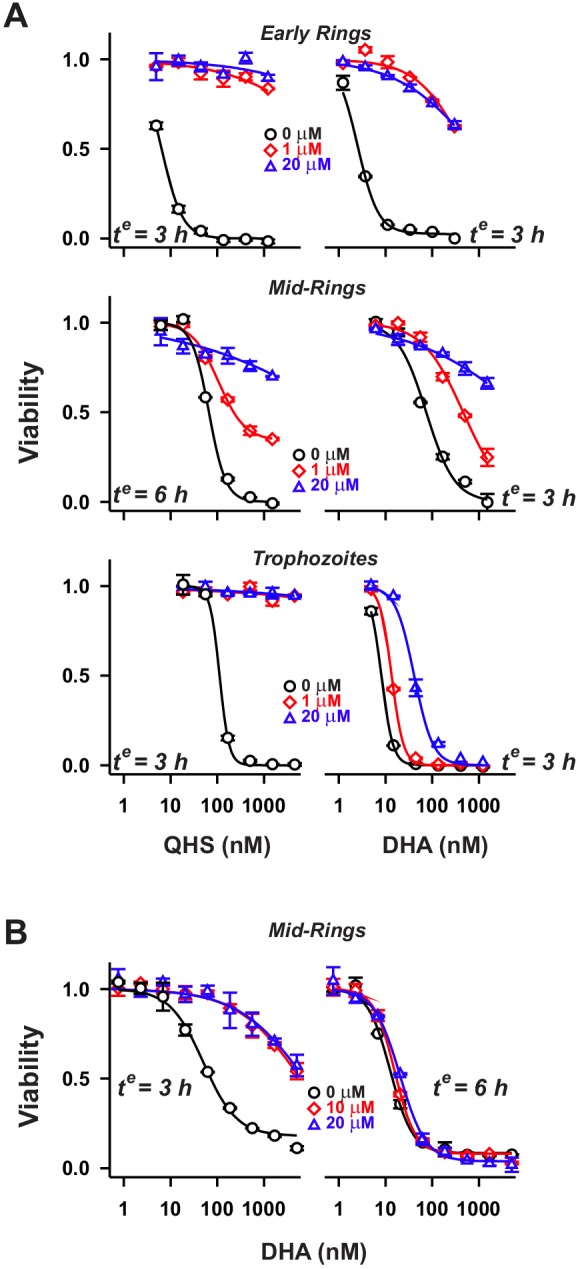
**Influence of cysteine protease inhibition on the activity of ARTs at different parasite stages.** (A) Parasites at early ring, mid-ring and trophozoite stages (1–2 h, 7–8 and 24 h post-invasion, respectively) were pre-treated with E64d at the concentrations indicated, and exposed to QHS or DHA for the exposure times (*t^e^*) indicated. Viability was assessed after 72 h. (B) Influence of exposure time on E64d antagonism of QHS activity in mid-ring stage parasites. Error bars correspond to the range of technical duplicates in a typical experiment. Results from a number of experiments are summarised in Fig. 4D (symbols).

A reversible cysteine protease inhibitor, N-acetyl-Leu-Leu-norLeu (ALLN), has been shown to antagonise the activity of short (4-h) drug pulses of DHA and QHS against trophozoites (Klonis et al., 2011), but ring stage parasites have not been examined. Given that ALLN is a relatively broad-range protease inhibitor (Francis et al., 1997), we examined the effects of E64d – a highly specific, epoxide-based, irreversible and cell permeable cysteine protease inhibitor that has been shown to inhibit haemoglobin degradation in *P. falciparum* (Rosenthal, 2004). As for the Fe chelators, the effect of E64d on activity of ARTs was dependent on the parasite stage, the antagonist concentration, the nature of the drug (i.e. DHA versus QHS) and on the duration of the drug pulse (Fig. 3). E64d (1 or 20 µM) completely abrogated QHS activity against trophozoites [>40-fold increase in LD_50_(3 h); Fig. 3A] but caused a more modest decrease in DHA activity (2- to 10-fold; Fig. 3A). This confirms the important role of haemoglobin degradation products as activators of ARTs at the trophozoite stage.

The degree of antagonism strongly depended on ART exposure time as evidenced by the potent antagonism of DHA activity in mid-rings subjected to a 3-h drug exposure [100-fold increase in LD_50_ (3 h)], and the almost complete absence of antagonism upon a 6-h exposure (Fig. 3B). When compared using the same exposure time, E64d antagonism of DHA activity was more marked in ring stage parasites compared to trophozoites (Fig. 3A, right-hand graphs). Surprisingly, early ring stage parasites exhibited the most pronounced antagonism [>100-fold increase in LD_50_ (3 h) with 1 µM E64d]. Taken together, these data indicate that cysteine proteases play a very important role in generating activators of ARTs, particularly in the early ring and trophozoite stages.

### Contribution of activators of ARTs at different stages

**Fig. 4.**
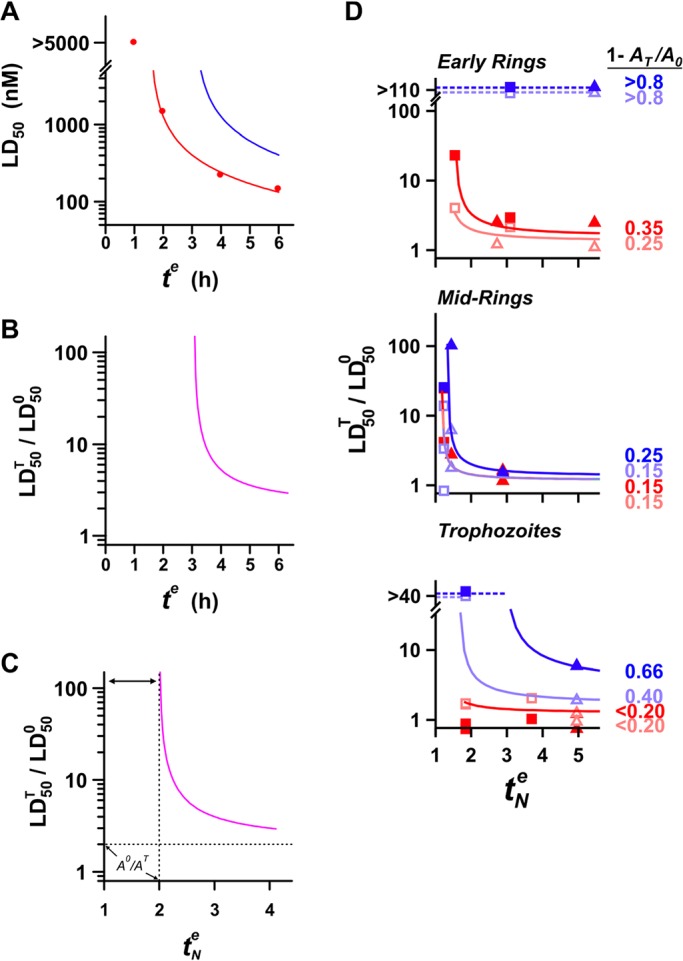
**Contributions of** Fe**-chelatable and E64d-inhibitable activators to the activity of ARTs at different parasite stages.** (A–C) Reduction of activator levels is predicted to produce exposure time-dependent changes in the degree of antagonism. The red symbols in A correspond to LD_50_ values for QHS against trophozoites at different exposure times. The red curve is the best fit obtained with the cumulative effective dose model with *K_m_=*390 nM and
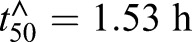
 (all red data are from Klonis et al., 2013b). The blue curve was constructed assuming a treatment produces a 50% reduction in activator levels (a doubling of 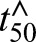 – see text for details). The LD_50_ ratios calculated in the absence and presence of treatment (superscripts 0 and T, respectively) from the red and blue curves are shown as a function of drug exposure time in B, and of the normalised exposure time in C. The region indicated by the double arrows in C corresponds to exposure times where 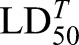 cannot be measured (*t^e^* is less than the 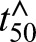 value for the treatment regime). (D) Activator levels at different parasite stages following chelator and protease inhibitor treatment. LD_50_ ratios were measured for QHS (squares) and DHA (triangles) at different drug exposure times following pre-treatment with BiPy (red) or E64d (blue). Measurements were performed at the lower (unfilled symbols) and the higher (filled) chelator and inhibitor concentrations indicated in Figs 2A and 3A and correspond to individual ratios measured from individual experiments. The 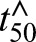 values used to calculate the normalised 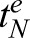 values are from Klonis et al., 2013b. Curves are calculated according to Eqn 3. The fractional reductions of activator induced by treatment [1*−*(*A_T_*/*A*_0_)], for the higher and lower inhibitor concentrations, are indicated to the right of the curves. The dashed blue curves correspond to 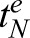 values where LD_50_ ratios cannot be evaluated.

The above results show that restriction of the level of activator antagonises ARTs in a manner that depends on the chelator or protease inhibitor concentration, the duration of ART exposure and intrinsic differences between QHS (lactone) and DHA (lactol). The interactions are further complicated by the complex dependence of activity of ARTs on stage, drug concentration and exposure time, even in the absence of chelators or inhibitors (Klonis et al., 2013b). An example of the dependence of QHS LD_50_ values on drug exposure time (*t^e^*) is shown in Fig. 4A (red symbols). We previously demonstrated that this complex dependence can be described by a cumulative effective dose model where parasite killing is related to the effective drug dose (*ED*) sensed by the parasite, which is a saturable function of the administered drug concentration, *D* (Klonis et al., 2013b). Given that ARTs require activation to exert their cytocidal activity, the effective dose likely reflects the production of activated drug, D*. The cell can be considered to contain an activator source (e.g. haemoglobin or cellular Fe uptake), an activator sink (e.g. haemozoin or Fe-binding proteins) and the activator, *A* (e.g. Fe or haem) capable of activating ARTs. Under steady-state conditions of activator, it can be shown that the production of activated drug (dD*/dt^e^) is a saturable process that follows Michaelis–Menten kinetics:
(1)
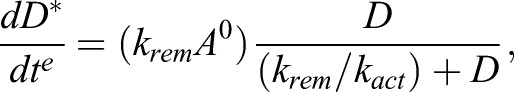
where *A*^0^ is the steady-state level of activator prior to the addition of drug, *k_rem_* is the first order rate constant for the cellular removal of *A* and *k_act_* is the second order rate constant for drug activation by the activator. Thus, *ED^max^* (or *k_rem_A*^0^, the maximum *ED*) reflects the level of activator and the cellular turnover of activator, whereas *K_m_* (or *k_rem_/k_act_*, the drug concentration producing half *ED^max^*) depends on the reactivity of the drug and the cellular turnover of the activator.

According to the cumulative effective dose model (Klonis et al., 2013b), the LD_50_ for a particular parasite stage depends on the drug exposure time according to:
(2)
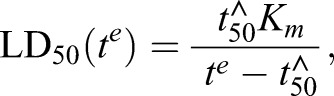
where 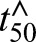 is the minimum exposure time required to kill 50% of the parasites (equivalent to 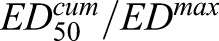 where 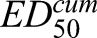 is the cumulative *ED* required to kill 50% of the parasites). Treatments that decrease activator levels will decrease *ED^max^* and increase the LD_50_ values measured at a particular drug exposure time. This effectively shifts the LD_50_ versus *t^e^* curves to longer times (Fig. 4A, blue versus red curve). As a result, the observed antagonism – defined as the ratio of LD_50_ values measured in the presence and absence of treatment – is strongly dependent on the time of exposure to ARTs with substantially less antagonism expected at the longer exposure times (Fig. 4B). This dependence on exposure time is strikingly evident in the measurements shown in Fig. 3B, which illustrate that mid-rings treated with 20 µM E64d exhibit an LD_50_ ratio of ∼100 with a 3-h DHA exposure, but a ratio of ∼1 (i.e. no antagonism) with a 6-h exposure.

From Eqn 2, it can be shown that a simple relationship exists between the LD_50_ value measured in the absence (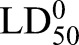) and presence (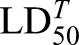) of a treatment that reduces the steady-state activator from level *A*_0_ to level *A_T,_* and the time of exposure to the ARTs:
(3)
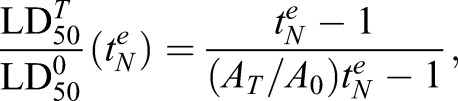
where 
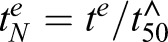
 represents the ART exposure time (*t^e^*) normalised by the 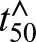 value in the absence of treatment (see Fig. 4C). Given that the 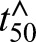 value is dependent on the drug used and on the parasite stage (Klonis et al., 2013b), the LD_50_ ratio becomes independent of the parasite stage and of drug chemical features (i.e. QHS versus DHA) when expressed as a function of 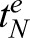, thus permitting quantitation of the fractional reduction in activator levels (1−*A_T_/A*_0_) induced by the treatment (Fig. S1D).

Ten-fold increases in BiPy (Fig. 4D, light versus dark red) or DFP (Fig. S1C, light versus dark green) concentrations produced only small additional reductions in the levels of activators of ARTs. This indicates close to complete chelation of the cellular Fe pool under the conditions used; this is consistent with the rapid membrane permeation and high Fe affinity of these chelators (Fig. 1C,D). The greatest chelator-mediated reduction in activity was 35% in early rings (Fig. 4D, red curves; Fig. S1C). This indicates that a maximum of ∼35% of the activity of ARTs in early rings can be ascribed to activation through the labile Fe pool. In mid-ring and trophozoite stages, the contribution of this chelatable Fe to the activation of ARTs is less than 20% (Fig. 4D, red curves; Fig. S1C).

By contrast, much larger reductions in activity were produced by haemoglobinase inhibition (Fig. 4D, blue). In early rings, even 1 µM E64d produced >80% reduction in activity, indicating that almost all activation of ARTs in early rings can be attributed to an E64-inhibitable activator. Trophozoites also exhibited large (up to 66%) E64d-concentration-dependent reductions in activity. Interestingly, mid-ring stages showed a much lower dependence on an E64d-inhibitable activator than the other stages, exhibiting a maximum reduction of activity of 25% with 20 µM E64d (Fig. 4D). This degree of antagonism was unaffected by halving the E64d concentration to 10 µM (Fig. 3B), indicating the E64d effects are saturated under these conditions. Thus, whereas all activity of ARTs in early rings and almost all activity (>80%) in trophozoites can be ascribed to activation by either chelator- or E64d-inhibitable activators, at most only ∼40% of the activity in mid-rings can be attributed to these activators. This indicates that additional modes of activation of ARTs, or activation-independent activity, are important in the mid-ring stage.

### Falcipains are the targets of the cysteine protease inhibitor, E64d, in ring stage parasites

**Fig. 5.**
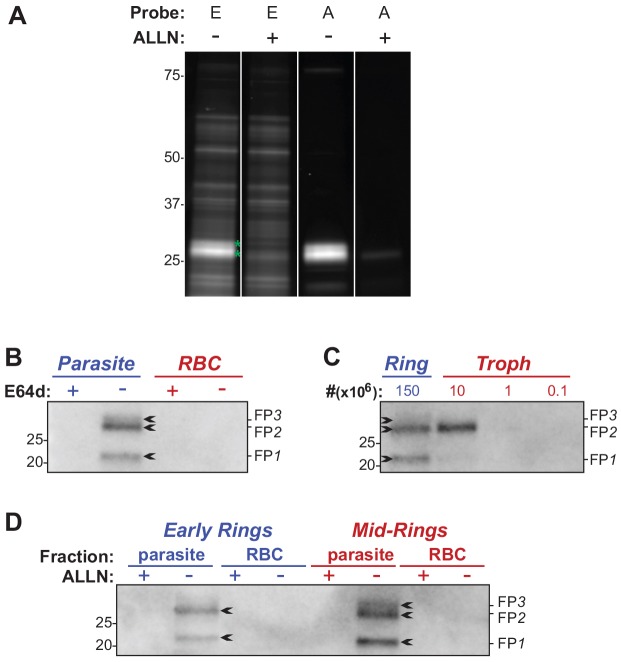
**Active falcipains are present in early ring stage *P. falciparum*.** (A) The acid derivative of Cy5–DCGO4 is a more specific label of cysteine proteases than the ester form. Parasite extracts from mid-rings (7–13 h post-invasion) were labelled with the acid (A) or ester (E) form of Cy5–DCG04 in the absence and presence of 10 µM ALLN and the labelled proteins (in the Triton X-100 supernatant) visualised. The lanes shown are from the same gel. Asterisks denote bands that were sensitive to ALLN inhibition. (B) Identification of E64d-inhibitable cysteine proteases in early rings. Parasite cultures (1–4 h post-invasion) were incubated for 3 h in the absence or presence of E64d, and the active cysteine proteases in parasite and host RBC fractions labelled with DCG04 acid and visualised following pulldown. The bands indicated by arrows represent FP1, FP2 and FP3 (see Table S1). (C) Falcipains detected in rings are not due to trophozoite contamination. DCG04-acid-labelled cysteine proteases were pulled down from ring and trophozoite-stage cultures with parasite numbers used indicated above the lanes. Analysis of Giemsa-stained smears of the ring stage culture indicated the sample loaded contained material from <2×10^5^ trophozoites (750 parasites counted with no trophozoites detected). (D) Levels of falcipains increase during ring stage growth. Labelling with DCG04 acid was performed as in B using tightly synchronised early ring (2–3 h post-invasion) and mid-ring (9–10 h post-invasion) parasites.

E64d is an epoxide ethyl ester that is hydrolysed *in situ* to release the active compound, which can react covalently with the active site of cysteine proteases. The biotinylated epoxide, DCG04 (Greenbaum et al., 2002), and its fluorescent analogue, Cy5–DCG04 (Stolze et al., 2012), have previously been used to identify active cysteine proteases in *P. falciparum*. Like E64d, these derivatives are esterified. We found that the Cy5–DCG04 ester labelled a number of proteins in detergent extracts of ring stage parasites (Fig. 5A, probe E), but that only a few of these were active cysteine proteases (i.e. ablated by pre-incubation with ALLN; Fig. 5A, asterisks). In contrast, the unesterified (acid) form of Cy5–DCG04 showed more specific labelling of active cysteine proteases (Fig. 5A, probe A). We therefore used the DCG04 acid to identify E64d-sensitive proteases in ring stage (4–7 h post-invasion) parasites. Infected red blood cells (RBCs) were lysed with saponin to generate fractions representing the parasite (saponin pellet) and the host RBC (haemoglobin-depleted saponin supernatant; see Materials and Methods). These samples were labelled with DCG04 acid and the biotinylated proteins visualised following western blotting. Three parasite-derived bands were observed, with no bands evident in the host RBC fraction (Fig. 5B). Pre-treatment of the cultures with E64d eliminated these bands (Fig. 5B), confirming that they represent the targets of E64d in rings.

**Fig. 6.**
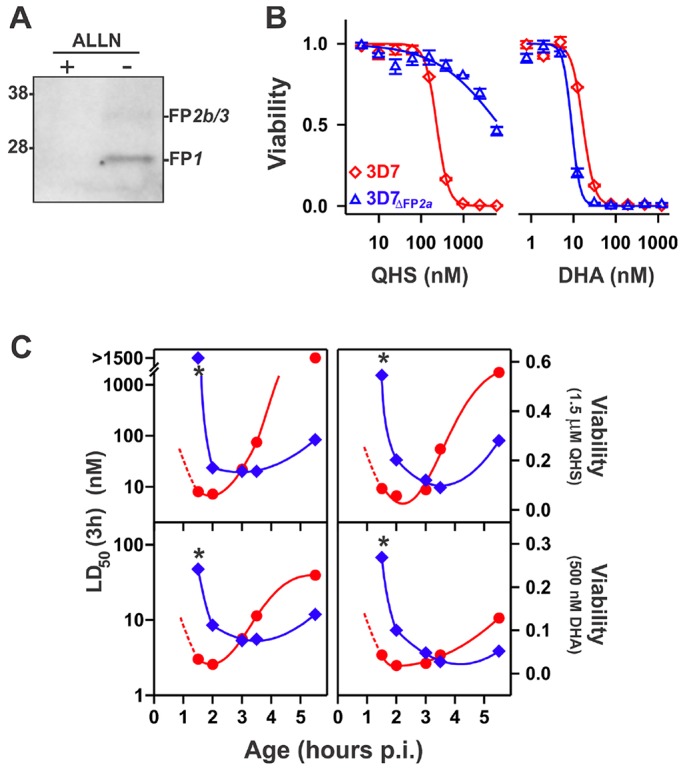
**Deletion of FP2a delays the onset of ART sensitivity in early rings.** (A) Active FP2a is not present in 3D7_ΔFP2a_ parasites. Active cysteine proteases were labelled with DCG04 acid in tightly synchronised early rings (2–4 h post-invasion) of the 3D7_ΔFP2a_ strain in the absence or presence of 10 µM ALLN. (B) Effect of FP2a knockout on the ART sensitivity of trophozoites*.* Tightly synchronised 3D7 and 3D7_ΔFP2a_ parasites were subjected to 3-h QHS or DHA pulses at the early trophozoite stage (25 h post-invasion). Viability was assessed after 72 h using flow cytometry. (C) Deletion of FP2a delays the onset of ART sensitivity. Tightly synchronised 3D7 (red) or 3D7_ΔFP2a_ (blue) parasites were subjected to 3-h drug pulses with QHS (top) or DHA (bottom) at different times post-invasion. Shown are LD_50_ (3 h) values and the viabilities measured at the highest ART concentrations. Parasite age (p.i., post-invasion) corresponds to the average parasite age at the start of each assay. The dashed portion of the red curve corresponds to the extrapolated behaviour of 3D7 parasites based on a previous study (Klonis et al., 2013b). Asterisks illustrate large differences in the drug sensitivities of the earliest ring stages examined.

In order to identify the cysteine protease targets, an early ring culture (1–7 h post-invasion) was scaled up and the biotinylated proteins were pulled down and analysed by mass spectrometry. The three top proteins were FP1 (PF3D7_1458000), FP2 [of which there are two forms, FP2a PF3D7_1115700 and FP2b (PF3D7_1115300)] and FP3 (PF3D7_1115400) (Table S2). The observed protein sizes (Fig. 5B) are consistent with the predicted molecular masses of the mature forms of these falcipains (20–30 kDa), and with previous studies (Greenbaum et al., 2002). To confirm the targeted proteins are indeed falcipains, pulldown experiments were performed using transfectants in which the FP1 and FP2a genes have been deleted (3D7_ΔFP1_ and 3D7_ΔFP2a_; Sijwali et al., 2004; Sijwali and Rosenthal, 2004). As expected, FP1 and FP2a, are absent in these parasites (see Fig. 6A; Fig. S3A).

Although FP1 has been detected in early rings, previous studies have suggested expression of FP2a and FP2b, and of FP3 is restricted to trophozoites (Greenbaum et al., 2002). To ensure that there was no trophozoite contamination, the ring stage cultures used for these studies were subjected to sorbitol treatment and passed twice through a magnetic column. A careful analysis of Giemsa-stained smears confirmed the level of trophozoite contamination in the ring cultures was more than 10-fold below the level of detection of trophozoite-derived falcipains (Fig. 5C). This demonstrates that the FP2 and FP3 signals indeed arise from early stage rings. Hence, it appears that FP1–FP3 are present in ring stage parasites and could be involved in activation of ARTs at this stage.

We next examined the pattern and levels of expression of the falcipains in early ring stages (1–2 h post-invasion) and mid-rings (8–9 h post-invasion) (Fig. 5D). The levels of active falcipains increase with parasite age, with FP3 becoming evident in mid-ring stage parasites. The equivalent bands were observed when Cy5–DCG04 was used to fluorescently label the E64d targets (Fig. S2). These data indicate that the higher sensitivity of early rings to ARTs compared to mid-rings is not due to higher expression of falcipains.

### FP2a plays a role in activation of ARTs in early rings

Having identified falcipains as the targets of E64d in rings, we used knockout and knockdown strategies to examine the roles of each of the four falcipains that are expressed in blood stage parasites, and which potentially have overlapping functions. Deletion of FP2b or FP1 did not affect the sensitivity of parasites to 3-h pulses of QHS or DHA in either rings or trophozoites (Fig. S3). In contrast, parasites carrying a deletion mutant of FP2a (3D7_ΔFP2a_) show reduced QHS sensitivity compared to wild-type parasites, at the early trophozoite stages (Fig. 6B), consistent with a previous report (Klonis et al., 2011). Interestingly, this reduction in sensitivity was not evident when DHA was used as the killing agent (Fig. 6B).

Deletion of FP2a produced a complex effect on drug sensitivity during the ring stage (Fig. 6C). At the early ring stage (1.5 h post-invasion) 3D7_ΔFP2a_ parasites were substantially less sensitive to QHS (>100-fold) and DHA (16-fold) than the 3D7 parent. This relative sensitivity was reversed in older rings (6 h post-invasion) where the 3D7_ΔFP2a_ strain exhibited increased sensitivity to QHS (18-fold) and DHA (3-fold). Examination of sensitivities across the ring stage suggests that this reflects a shift in the window of early ring hypersensitivity towards longer times in the 3D7_ΔFP2a_ strain. Given that the growth rates of 3D7 and 3D7_ΔFP2a_ parasites are very similar (Sijwali and Rosenthal, 2004), this shift in the sensitivity window is unlikely to be caused by a delay in the development of 3D7_ΔFP2a_ and suggests that FP2a is responsible for providing the activators of ARTs in early rings, with other activators and targeting mechanisms becoming available from about 3 h post-invasion

The decreased sensitivity of early ring stage 3D7_ΔFP2a_ resulted in a substantial increase in the number of parasites that survive short pulses of high concentrations of ARTs (Fig. 6C, right panels). The observation that 26% of parasites survive a pulse of 500 nM DHA is particularly important as parasite survival under these conditions has recently been shown to directly correlate with resistance to ARTs in the field (Dogovski et al., 2015; Witkowski et al., 2013). The data indicate that FP2a deletion renders the parasite partially resistant to pharmacologically relevant levels of DHA.

### Knockdown of FP3 does not affect parasite growth but contributes to ART sensitivity in early rings

FP3 is considered the only essential (blood stage) enzyme in the falcipain family as it has proven resistant to genetic deletion (Sijwali et al., 2006). Here, we used an inducible *glmS* ribozyme system to conditionally knockdown FP3 in 3D7 and 3D7_ΔFP2a_ strains, generating the parasite lines 3D7_FP3kd_ and 3D7_ΔFP2a/FP3kd_. In the presence of the cofactor glucosamine (GlcN), the *glmS* ribozyme degrades the mRNA encoding the targeted gene, thereby reducing its expression (Prommana et al., 2013). The GlcN-inducible *glmS* ribozyme and a 3×HA tag were incorporated into the 3′ untranslated region (UTR) of the FP3 gene by homologous recombination (Fig. S4A). Correct integration of the *glmS* plasmid was confirmed by PCR.

**Fig. 7.**
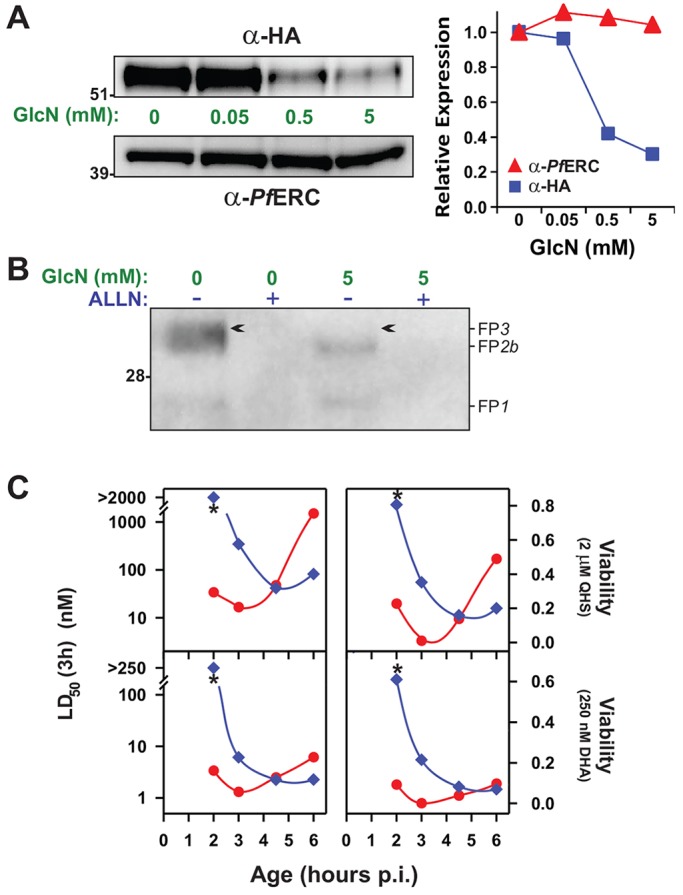
**Knockdown of falcipain-3 contributes to a resistance phenotype in early rings.** (A) FP3 is knocked down using the *glmS* ribozyme system. 3D7_ΔFP2a/FP3kd_ rings [0–20 h post-invasion (p.i.)] were incubated with increasing concentrations of GlcN for 20 h. Saponin-treated trophozoite extracts were subjected to western blotting and probed with anti-HA or -*Pf*ERC antibodies and the protein levels quantified by densitometry. (B) Knockdown of FP3 activity*.* Parasite fractions derived from trophozoites subjected to 0 or 5 mM treatment with GlcN (see A) were labelled with DCG04 acid in the absence or presence of 10 µM ALLN. (C) Knockdown of FP3 in a ΔFP2a background leads to an early ring resistance phenotype. Cultures containing 3D7_ΔFP2_ (red symbols) or 3D7_ΔFP2a/FP3kd_ (blue) parasites were treated with 2.5 mM GlcN (30 h), synchronised, and then subjected to 3-h drug pulses with QHS (top) or DHA (bottom) at different times post-invasion. Shown are LD_50_ (3 h) values and the viabilities measured at the highest ART concentrations. Parasite age corresponds to the average parasite age at the start of each assay. Asterisks illustrate large differences in the drug sensitivities of the earliest ring stages examined.

FP3 has an N-terminal pro-domain (involved in trafficking to the digestive vacuole) and a C-terminal mature domain (containing the active site) (Subramanian et al., 2007). The HA-tagged protein exhibited an apparent molecular mass of 60 kDa in both the 3D7_FP3kd_ (Fig. S4B) and 3D7_ΔFP2a/FP3kd_ (Fig. 7A) strains, as anticipated for full-length FP3. Consistent with a previous study in which FP3 was tagged at the C-terminus (Sijwali et al., 2006), only full-length FP3 was observed using the anti-HA antibody. The failure to detect a band corresponding to mature (active) FP3 (expected molecular mass of ∼30 kDa), indicates that in addition to N-terminal processing, C-terminal processing (which removes the tag) occurs as part of the formation of mature FP3.

Treatment of ring stage 3D7_ΔFP2a/FP3kd_ parasites with 5 mM GlcN for 20 h, resulted in efficient FP3 knockdown (70–75%; Fig. 7A). By contrast, the level of a control protein, *P. falciparum* ERC (*Pf*ERC), remained unchanged, indicating that the treatment did not retard parasite growth. Moreover, when maintained for 4 days in the presence of 5 mM GlcN, 3D7_ΔFP2a/FP3kd_ and wild-type 3D7 parasites developed at the same rate. To confirm that the knockdown also affected active FP3, we labelled parasite extracts with the DCG04 acid. In the absence of GlcN-induction, a broad, ALLN-inhibitable 30-kDa band was pulled down from 3D7_ΔFP2a/FP3kd_ parasites (Fig. 7B*,* arrow). Based on protein migration profiles observed in wild-type (3D7) parasites, the top and bottom parts of this band likely represent FP3 and FP2b. The FP3 band was lost upon GlcN induction (Fig. 7B) confirming knockdown of FP3 activity. Similar behaviour was observed in the 3D7_FP3kd_ strain (Fig. S4C).

We were surprised by the fact that parasite growth was not affected by the significantly decreased FP3 expression levels, even in the background of disrupted FP2a. We did note that the 3D7_ΔFP2a/FP3kd_ parasites exhibited a swollen digestive vacuole phenotype at early trophozoite stage, but only at a level equivalent to that observed for the FP2a-knockout parent. These results indicate that even a low level of FP3 activity along with the minor isoform FP2b is sufficient to sustain haemoglobin degradation at a level that permits normal growth. Extensive functional redundancy is known to operate between the falcipains and also with the plasmepsins, the other major family of haemoglobinases (Drew et al., 2008; Lin et al., 2015).

We investigated whether concomitant knockdown of FP3 in a background of disrupted FP2a would further modulate drug sensitivity. Knockdown of FP3 shifted the window of hypersensitivity even further than was observed for 3D7_ΔFP2a_ (Fig. 7C). As no growth effects were observed upon FP3 knockdown, this further delay in the window of QHS and DHA sensitivity does not reflect differences in parasite ages or growth rates. Importantly, this additional shift further increases the number of early ring stage parasites that can survive pharmacologically relevant DHA doses and extends the age to which rings can survive to 3 h post-invasion. These results strongly indicate that FP3 is involved in the potent activity of ARTs at the early ring stage and that loss of both FP2a and FP3 activity can lead to a phenotype that approaches that observed in resistant parasites from the field.

## DISCUSSION

Current thinking suggests that the mostly likely activators of ARTs are reduced haem and/or free Fe^2+^ (Klonis et al., 2013a). Here, we confirm that membrane-permeable Fe chelators can antagonise the activity of ARTs to a small degree in trophozoites (Fig. 2, Fig. S1), consistent with a previous report (Stocks et al., 2007), and show they produce a greater, though still limited, degree of antagonism in early rings (Fig. 2; Fig. S1). A quantitative analysis of this antagonism demonstrates that ∼35% of the activity of ARTs in early rings, and ∼15% of the activity in trophozoites can be attributed to chelatable Fe-mediated activation of ARTs (Fig. 4; Fig. S1). This indicates that alternative activators of ARTs must operate at both the trophozoite and the ring stage. In the case of trophozoites, the bulk (>65%) of the activity of ARTs can be attributed to an E64d-inhibitable activator (Fig. 4), likely representing haem generated during haemoglobin digestion (Klonis et al., 2011).

Surprisingly, almost all the activity of ARTs in early rings (>80%) can also be ascribed to an E64d-inhibitable activator (Fig. 4). Haemoglobin digestion is most active in the trophozoite stage, and it is generally assumed that little haemoglobin uptake occurs in the youngest ring stage parasites (see Abu Bakar et al., 2010 and references therein). However, a single inactive cytostomal ring is present at the periphery of merozoites (Hanssen et al., 2010), indicating that each daughter cell brings its own feeding apparatus upon invasion of a new RBC, and *P. falciparum* CRT, a marker of the digestive vacuole has been detected in early rings (Gligorijevic et al., 2008). Moreover micropinocytosis has been observed from the parasite surface (Slomianny, 1990) in early stage parasites, and fluorescence and electron tomography studies have shown that, by the mid-ring stage, parasites have detectable acidified compartments that contain small haemozoin crystals (Abu Bakar et al., 2010; Grüring and Spielmann, 2012). Taken together with the evidence for expression of active FP2 and FP3 (Fig. 5), this suggests that haemoglobin degradation occurs much earlier than generally recognised and that the E64d-inhibitable activator in early ring stage parasites is haemoglobin degradation-derived haem.

Unlike in the other stages, the chelatable Fe and haemoglobin-derived haem account for only a fraction (∼40%) of the activity of ARTs in mid-ring stages (Fig. 4). This suggests other activators or other mechanisms of drug action might contribute to the activity of ARTs and might play a particularly pronounced role during this parasite stage. For example, cofactors involved in maintaining redox homeostasis (Haynes et al., 2012) and mitochondrially located haem-containing components (Wang et al., 2010) have been proposed to be activators of ARTs. Alternatively unactivated ARTs could directly inhibit targets, as has recently been proposed for the *P. falciparum* phosphatidylinositol-3-kinase (*Pf*PI3K) (Mbengue et al., 2015).

We previously demonstrated that parasites exhibit quite different responses to QHS and DHA with respect to the onset of killing (Klonis et al., 2013b). These variations reflect different 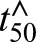 values (2- to 3-fold greater for QHS across all intraerythrocytic stages) and manifest as an increased lag time before killing is initiated with QHS. Differences in 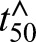 values have important implications for predicting the *in vivo* behaviour of different ARTs, and new synthetic endoperoxides, such as OZ439 (Charman et al., 2011), as both the drug pharmacokinetics (exposure time) and the underlying potency of the drug (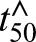) will influence the promptness of action. As evident in the present study, the 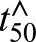 values and the drug exposure time also affect the degree of drug interactions (Eqn 3) and explain why, for a given exposure time, chelators and inhibitors antagonise QHS activity to a greater extent than DHA activity across all parasite stages (Figs 2 and 3). This also accounts for the observation that early ring and trophozoite 3D7_ΔFP2a_ parasites exhibit pronounced differences in sensitivity to QHS compared to wild-type parasites, but less differences in DHA sensitivity (Fig. 6).

Pulldowns with biotinylated E64d demonstrated expression of the full suite of falcipains in ring stages (Fig. 5). FP1 is the most divergent of the falcipain family and is reported not to be involved in haemoglobin digestion (Greenbaum et al., 2002). As expected, deletion of FP1 had no effect on parasite sensitivity to ARTs (Fig. S3). Although FP2b is a haemoglobinase, we similarly found its deletion had no effect on parasite sensitivity (Fig. S3), which might reflect its known redundancy with FP2a (Sijwali et al., 2006). In contrast, disruption of FP2a and FP3 activity had pronounced effects on sensitivity to ARTs in early rings (where almost all the activity of ARTs is E64-inhibitable in the wild type; Fig. 7). Disruption of both FP2a and FP3 activity enabled early rings to survive a physiologically relevant DHA dose (Fig. 7) at a level similar to that observed in ART-resistant field strains (Dogovski et al., 2015).

It is interesting to consider the factors that determine the efficacy of the action of ARTs in early rings. The rate of parasite killing will depend on the flux of activation of ARTs, which will be influenced both by the rate of haemoglobin uptake and the level of falcipain activity. Sensitivity to ARTs will also depend on the ability of the parasite to defend itself against cellular damage, which recent work indicates involves a stress response that engages the ubiquitin–proteasome system (Dogovski et al., 2015). ART resistant parasites exhibit a high level of resistance at the early ring stage (Witkowski et al., 2013), a phenotype that has been linked to mutations in the K13 propeller protein (Ariey et al., 2014; Miotto et al., 2015), and recent reports provide evidence that mutations in K13 are associated with an up-regulated cell stress response (Dogovski et al., 2015; Mok et al., 2015). We propose that the high susceptibility of early rings derives from active haemoglobin degradation and, thus, haem production, prior to the onset of mechanisms for repairing damage induced by activated ARTs.

Accordingly, knockout of FP2a and knockdown of FP3 in an FP2a-knockout background in 3D7 parasites results in an ART-resistant phenotype that manifests at the early ring stage (Fig. 7). Our data thus shows that a decreased level of activators of ARTs in early rings could make an important contribution to ART resistance. Although there is, to date, no evidence for altered falcipain expression profiles in ART-resistant field parasites, there is genetic evidence suggesting a role for additional parasite factors that augment K13-mediated resistance in field isolates (Straimer et al., 2015). Interestingly, a mutation in FP2a was generated by subjecting an ART-sensitive F32-Tanzania clone to escalating pressure with QHS (Ariey et al., 2014). It might be that large reductions in falcipain activity are too costly with respect to parasite fitness during infections *in vivo* to represent a dominant mechanism for generating resistance in the field, but stage-specific down-modulation of activity could enhance the effectiveness of K13 mutations. Further work is needed to determine whether alterations in the timing of expression of particular falcipains contribute to high level resistance in field strains.

## MATERIALS AND METHODS

### Materials

Artemisinin (QHS), dihydroartemisinin (DHA), 2,2′-bipyridyl (BiPy), deferiprone (DFP), E64d, ALLN and glucosamine (GlcN) were purchased from Sigma-Aldrich; Syto-61 and calcein-AM were from Life Technologies; streptavidin–agarose was from GenScript; protease inhibitor cocktail was from Roche; streptavidin–horseradish-peroxidase (HRP) conjugate and the BCA Protein Assay Kit were from Pierce.

The acid and ester forms of DCG04 (Greenbaum et al., 2000) were prepared by conjugating an epoxide group to the peptide NH_2_-Leu-Tyr-Ahx-Lys(Biotin)-CONH_2_ (where Ahx is an aminohexanoate linker). Diethyl (2S,3S)-(+)-2,3-epoxysuccinate (Thermo Fisher Scientific) was subjected to partial hydrolysis to its mono-acid ester derivative by a 4-h treatment with 1 equivalent of KOH in ethanol at 4°C, followed by an ethyl acetate extraction and hydrochloric acid work-up (based on a protocol reported by Bogyo et al., 2000). The resulting mono-acid ester derivative (5–10 equivalents) was conjugated to the free N-terminus of the peptide in dimethylformamide by incubation for 1 h in the presence of a 5–10-fold molar excess of N,N′-diisopropylcarbodiimide. Limited ester hydrolysis during the incubation results in the production of the epoxide ester as well as the hydrolysed (epoxide acid) forms of the conjugates. These products were purified on an Agilent 1100 HPLC using a 21.2×150 mm×5 m Varian C18 column. A linear gradient of 0–60% buffer B over 30 min was employed, where buffer A was 0.1% trifluoroacetic acid in water and buffer B was 0.1% trifluoroacetic acid in acetonitrile, with the peaks detected at a wavelength of 220 nm. All pure fractions were lyophilised on a Virtis Benchtop SLC freeze dryer. Electrospray ionisation mass spectrometry (ESI-MS) analysis was performed on an Agilent 6220 Accurate Mass TOF. DCG04 ester (Yield 20%; Purity >90%; MW: Th. 903.1, Obs. 903.5). DCGO4 acid (Yield 10%; Purity >95%; MW: Th. 875.0, Obs. 875.4).

Cy5-DCG04 was provided by Wouter van der Linden and Matt Bogyo, Stanford University, CA. The acid form was prepared by incubating Cy5–DCG04 in 10 mM NaOH and 10% DMSO for 1 h prior to its use in labelling reactions.

### Characterisation of Fe chelators

Stock solutions of BiPy (500 mM) were prepared in DMSO. Stock solutions of DFP (500 mM) were prepared in water by adding concentrated HCl dropwise until the DFP was completely dissolved. Chelation of Fe^2+^ by Fe chelators was measured spectrophotometrically by monitoring the change in absorbance of chelator solutions upon addition of FeSO_4_. Measurements were performed in 20 mM MES, pH 5. The effect of Fe chelators on Fe^2+^-mediated degradation of QHS was determined in 50% acetonitrile and 50% water containing 10 mM MES, pH 5 by monitoring the time-dependent loss of QHS by LCMS (Creek et al., 2005). The chelators (20 mM) were either pre-incubated with FeSO_4_ (3 mM) prior to the addition of QHS (30 µM) or were added immediately after the QHS was added to the FeSO_4_.

For live-cell imaging of Fe chelator-treated parasites, ring (5–15 h post-invasion) and trophozoite (28–38 h post-invasion) stage parasites were prepared by sorbitol treatments as described previously (Adisa et al., 2003). Parasite-infected RBCs were labelled overnight with BODIPY-TR-ceramide (Adisa et al., 2003), then with calcein-AM (2 μM) for 1 h under standard culture conditions. Parasites were then immobilised as a monolayer in a closed micro-chamber (iBidi) coated with erythroagglutinin PHA-E, the medium replaced with fresh medium containing chelators (100 µM), and incubated for 15 min (BiPy) or 30 min (DFP) at 37°C prior to collection of images using a DeltaVision Elite fluorescence microscope (Applied Precision). Controls containing no added chelator were also prepared and measured prior to the chelator-treated samples to avoid time-dependent leakage of calcein from the parasites biasing the chelator-dependent increases in intensity that were being measured. Images were processed and analysed using NIH ImageJ (http://imagej.nih.gov/ij/). The fluorescence intensity of calcein within the parasite compartment was quantified and used to measure the effect of chelator treatment on the parasite calcein signal.

### Culturing of parasites and assessment of viability

The wild-type parasite strain 3D7 and the falcipain deletion mutants 3D7_ΔFP1_ (Sijwali et al., 2004), 3D7_ΔFP2a_, and 3D7_ΔFP2b_ (Sijwali and Rosenthal, 2004) were cultured as described previously (Xie et al., 2014). Drug pulse assays were performed with tightly synchronised parasite cultures (1–3 h age window; Xie et al., 2014), and parasite viability was determined in the cycle following the drug pulse by analysing SYTO 61-stained cultures by flow cytometry (Klonis et al., 2011). Viability is defined as the fraction of parasites that survive drug treatment and are able to enter the next parasite cycle. LD_50_ is defined as the drug concentration producing 50% loss of viability and was determined by fitting a sigmoidal function to the dose–response profiles. The LD_50_ value is a direct measure of killing and is independent of growth retardation effects and signal contributions from gametocytes (Paguio et al., 2011; Xie et al., 2014). For examination of the effects of treatment with Fe chelators and cysteine protease inhibitors, synchronised parasite cultures were pre-treated with the chelator or inhibitors for 0.5 to 1 h prior to the addition of QHS or DHA. Controls containing no added chelators or inhibitors were always prepared at the same time as the treated samples, and were used to compare the effects of the added chelators and inhibitors and to calculate LD_50_ ratios. Using this approach, we can confidently measure 2-fold increases in LD_50_ values due to treatment. For treatments producing LD_50_ values that were outside the range of drug concentrations used in the assays, the LD_50_ ratio is reported as ‘
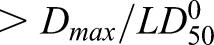
’, where *D_max_* is the maximum drug concentration used.

### DCG04 acid pulldown of cysteine proteases

Cultures were synchronised to a tight window (ranging from 1 to 6 h, depending on the experiment) by sorbitol treatment, plus two magnetic column treatments, as previously described (Xie et al., 2014) in order to minimise contamination of other stages. Aliquots of synchronised culture (2×10^8^–10×10^8^ parasites) were lysed by saponin (0.025%, w/v) and the parasite pellet was collected by centrifugation (53,500* **g***, 10 min, 4°C, pH 7.2). Samples were divided into parasite and RBC lysate fractions. An equal volume of Ni-NTA beads was incubated with the RBC lysate fraction for 10 min to deplete haemoglobin (Ringrose et al., 2008). Triton X-100 (0.2% v/v, pH 5.5) was added to both parasite and RBC fractions. DCG04 acid (1 µM) was added for 30 min at room temperature (pH 5.5), then the reaction was stopped by adding an equal volume of 1% SDS, 10 mM DTT, 1 mM TCEP, 2.5 mM EDTA and 1× protease inhibitor cocktail, pH 7.2. Pulldown was performed with 25 µl streptavidin–agarose for 1 h. Beads were washed with 1% SDS and proteins were eluted with 6 M urea in 1× SDS-PAGE sample loading buffer. Protein eluates were subjected to SDS-PAGE and western blotting, and probed with streptavidin–HRP (1:2500). For protein identification, on-bead trypsin digestion was carried out overnight. The digestion products were analysed by LC-MS/MS using Orbitrap Elite (Thermo Fisher). Protein hits were identified using the Mascot search engine with the UniProt database. For negative controls, parasite cultures were incubated with E64d (10 µM) for 3 h prior to saponin lysis, or lysates were incubated with ALLN (10 µM) for 15 min prior to the addition of DCG04 to compete for the targets.

### Cy5–DCG04 labelling of cysteine proteases

Cultures containing synchronised early rings (2×10^8^–5×10^8^ parasites) were lysed with saponin (0.05%, w/v) and the pellet was solubilised with Triton X-100 (0.1%, v/v). The negative control sample was incubated with ALLN (10 mM) for 30 min. Samples were then centrifuged (16,060* **g***, 10 min and 4°C) and soluble and insoluble fractions collected and incubated with Cy5–DCG04 (1 µM) for 1 h at room temperature (pH 5.5). The protein content in each sample was determined by a BCA protein assay. An equal amount of each sample (same protein content) was subjected to SDS-PAGE and protein bands were visualised using a Typhoon Fluorescence scanner (GE Healthcare).

### FP3 knockdown by *glmS* ribozyme

The HA-*glmS* plasmid harbouring the dihydrofolate reductase (DHFR) selection cassette (Elsworth et al., 2014) was modified to remove the stop codon and the FP3 gene was inserted using *Sal*I and *Pst*I; and confirmed by sequencing. For 3D7_ΔFP2a/FP3kd_ transfectants, an HA-*glmS* plasmid carrying a puromycin N-acetyl-transferase (PAC) selection cassette was employed. FP3-HA-*glmS* (100 µg) were used to transfect 3D7 and 3D7_ΔFP2a_ parasites. Transfectants were selected with 5 nM WR99210 (3D7_ΔFP2a_) or 100 ng/ml puromycin (3D7_ΔFP2a/FP3kd_). They were cycled on and off the drug to select for integration into the FP3 locus. Clones were obtained by limiting dilution and integration was confirmed by PCR.
